# The Effect of Free and Nanoliposomal Curcumin on the Viability and Acid Production of Single‐Species (*Streptococcus mutans*) and Polymicrobial Biofilms

**DOI:** 10.1002/cre2.70290

**Published:** 2026-02-17

**Authors:** Parisa Habibi, Farideh Tabatabaei Yazdi, Seyed Ali Mortazavi, Mohammad Morad Farajollahi

**Affiliations:** ^1^ Department of Food Science and Technology, Faculty of Agriculture Ferdowsi University of Mashhad Mashhad Iran; ^2^ Department of Medical Biotechnology, Faculty of Allied Medicine Iran University of Medical Sciences Tehran Iran

**Keywords:** biofilm, curcumin, nanoliposome, *S. mutans*

## Abstract

**Objectives:**

Dental caries is a global health concern caused by the formation of cariogenic biofilms and their acid production. While various approaches have been tried, the rise of antibiotic‐resistant microorganisms necessitates safer and more effective antimicrobial solutions. Natural substances can be safe and effective alternatives in controlling dental biofilms and preventing tooth decay. In this study, the effect of free and nanoliposomal curcumin on oral biofilms (single species and polymicrobial) was investigated.

**Material and Methods:**

Curcumin nanoliposomes were prepared using the thin layer hydration method, and their characteristics (size, zeta potential, and release of curcumin from liposomes) were evaluated. An Active Attachment model was used to compare the effect of free and nanoliposomal curcumin in the single‐species (*Streptococcus mutans*) and poly microbial biofilms and amount of acid production by them.

**Results:**

According to the obtained results, the diameter of liposomes was 98.83 ± 2.89 nm, and the release of curcumin from liposomes was less than 50% after 24 h. The MIC of free and non‐liposomal curcumin against *S. mutans* was 250 and 222 μg/mL, respectively. Nanoliposomal curcumin reduced the number of cells in polymicrobial and single‐species biofilms and acid production by them.

**Conclusions:**

Due to the results obtained in this research, nanoliposomal curcumin had good antibacterial, anti‐biofilm, and acid production inhibition properties. Maybe nanoliposomal curcumin can be used in oral and dental health products.

## 1 Introduction

Throughout life, tooth decay may occur in both primary (baby) and in permanent teeth and damage the tooth crown and expose the root surfaces. Dental caries refers to a complex disease caused by biofilms generated by frequent consumption of fermentable substances, carbohydrates (mainly free sugars), poor oral hygiene, as well as insufficient use of fluoride (Pitts et al. 2017).

There are two forms of bacteria in the mouth: planktonic and biofilm. Biofilms are organized microbial communities encapsulated and enclosed by an exopolysaccharide (EPS) matrix (Abebe 2021; Bortolaia and Sbordone 2002; Jhajharia et al. 2015). They form mainly on surfaces such as teeth, dentures, and implants (Teughels et al. 2006), as well as in sites including pits and fissures of teeth (Gurenlian 2007; Metwalli et al. 2013), tongue, gingival sulcus, palates, tonsils (Abebe 2021), gums (Sbordone and Bortolaia 2003), and root canals (Jhajharia et al. 2015).

In addition to acid tolerance, organic acid production and biofilm formation represent the main invasive characteristics associated with *Streptococcus mutans*. *S. mutans* adhere to salivary pellicles on teeth by glucans and glucan‐binding proteins which in addition to building the structure of the growing biofilm provide additional attachment sites for planktonic cells (Scharnow et al. 2019). Lactic acid is the organic acid produced from dietary sugars by *S. mutans* biofilms. Acid accumulation in biofilms results in a decrease in pH, in turn leading to the relative dissolution of calcium and phosphate (demineralization) in the surface layer of the tooth (Pitts et al. 2017). The tooth decay process begins under the enamel surface by demineralization and organic acids made by bacteria in biofilms and sugar metabolism. The decay progression may result in cavities in the tooth enamel and damage the dental pulp, finally leading to tooth extraction or root canal treatment (Amissah et al. 2021). Curcumin is an orange–yellow powder that is part of the spice turmeric (*Curcuma longa*) from the Zingiberaceae family, natively growing in India and Southeast Asia (Omosa et al. 2017). Curcumin is a bioactive substance that belongs to natural phenols (Praditya et al. 2019). Researchers report that curcumin has a wide range of beneficial properties, such as anti‐tumor, antioxidant, antiviral, anti‐inflammatory, antibacterial, and antifungal properties, owing to these properties, it has the potential to help manage various diseases such as asthma, diabetes, arthritis, allergies, arteriosclerosis, neurological diseases, and cancer. Due to the side effects of synthetic antibiotics, natural bioactive substances, such as curcumin obtained from plant products, could be a good substitute for them (Praditya et al. 2019; Gupta et al. 2013).

In a systematic review paper, Ehteshami et al. (2021) indicated curcumin's promising antibacterial and anti‐caries effects, including inhibition of *S. mutans* growth, acid production, and ATPase, as well as sortase A activities. Curcumin has been introduced as a natural, available, safe, and cheap agent to enhance oral and dental health (Ehteshami et al. 2021).

Despite curcumin's beneficial properties, its formulation faces many challenges, given its poor physical properties. It is a hydrophobic molecule with relatively low water solubility (Kharat and McClements 2019; Tønnesen et al. 2002). Besides its extensive systemic metabolism, curcumin's poor solubility may explain its limited bioavailability (Sharma et al. 2004). Strategies like liposomes and complexes with phospholipids and cyclodextrins, along with solid dispersions, have been developed to enhance their bioavailability (Kakkar et al. 2011). Curcumin's numerous fundamental properties with potential applications in different fields can be enhanced by liposomal storage (Dogra et al. 2015). Liposomes are typically composed of phospholipids, generally separated from plant or animal cell membranes. Phospholipids are amphiphilic in nature, easily forming bilayers when dispersed in an appropriate solvent like water (T. M. Taylor et al. 2005). Liposomes are able to trap hydrophobic materials in lipid bilayers and hydrophilic ones in the aqueous core. Being biocompatible, they may improve the solubility of the material, besides slowly releasing them over time. Given these improved properties, liposomes may enhance the effectiveness of treatment (Amissah et al. 2021). In order to deal with *S. mutans*, Palmas et al. (2020) suggested using liposomes to improve the antimicrobial properties of essential oils or treating tooth decay and recommended their inclusion in mouthwash formulations to prevent tooth decay. Liposomal curcumin has been reported by an in vivo study to have greater bioavailability and faster absorption compared to crystalline curcumin (Takahashi et al. 2009). Furthermore, nanotechnology‐based drug delivery systems are rapidly developing owing to their ability to transfer larger volumes of therapeutic agents with poor solubility in water. This feature enables the use of drugs with limited value and application due to their poor solubility in water (Amissah et al. 2021).

The present study investigated the inhibitory activity of nanoliposomal curcumin on polymicrobial and *S. mutans* biofilms compared to the free form. The amount of acid production by these biofilms was also investigated.

## 2 Material and Methods

### 2.1 Bacterial Strains and Cultivation Conditions

*S. mutans* C180‐2 (clinical isolate) (De Stoppelaar et al. 1969) was cultivated using brain heart infusion (BHI) in anaerobic conditions (90% N_2_, 5% H_2_, and 5% CO_2_) at 37°C and pH 7.0. Saliva used for the polymicrobial biofilm assay was collected from one of the authors, a healthy adult volunteer, with full informed consent. To avoid cold damage during storage, two‐fold diluted saliva with glycerol (60%) transferred to a −80°C freezer (Exterkate et al. 2010).

The Mc Bain medium was used with some changes for the polymicrobial biofilms. This culture medium contained 2.0 g/L Bacto‐Peptone (Difco 0118‐01‐8), 2.5 g/L Menosin (Sigma M‐2378), 1.0 g/L yeast extract (Bacto 212750), 2.0 g/L peptone trypticase (BBL 211921), 0.35 g/L NaCl, 0.2 g/L CaCl_2_, 0.2 g/L KCl, 0.001 g/L Hemin (Sigma H‐1652), 0.0002 g per liter of vitamin K1 (McBain et al. 2005) with 0.2% sucrose and 50 mmol/L PIPES buffer at pH 7.0.

### 2.2 Liposomes Preparation

Liposomal curcumin was prepared by the thin layer evaporation technique. The lipid phase consisting of cholesterol (Sigma 57885), phospholipid (Lecithin‐ Sigma‐ 429415), vitamin E (Sigma 10191410), and Tween 80 (Sigma 9005656) was dissolved in ethanol (98%) containing curcumin (gifted from Dineh Company, Qazvin, Iran). Rotary under vacuum was used to remove ethanol and form a thin film. The thin film produced in the flask was hydrated with phosphate‐buffered saline. The formed film was subjected to nitrogen treatment to remove the solvent completely (Mourtas et al. 2011; M. Taylor et al. 2011). To reduce the size of liposomes to the nanoscale, ultrasound (VCX 750, USA) for 10 min was used (Rasti et al. 2012). A micro‐pore filter (0.22 μm) was used to separate curcumin nanoliposomes.

### 2.3 Properties of Nanoliposomal Curcumin

The absorbance of different concentrations of curcumin was read at 420 nm, and the concentration–absorbance standard curve was drawn. After drawing the standard curve, the concentration calculation equation based on absorption was obtained (Equation 1).

(1) $$\text{OD}\;=0.0454\times \;C_{\text{cu}}+0.006$$

In the above equation, *C*_cu_ is equivalent to curcumin concentration.

### 2.4 Encapsulation Efficiency

The liposomes were centrifuged using Amicon 10 kDa filters at 1400 × *g* for 12 min (Vergara and Shene 2019; Xu et al. 2012). The concentration of total curcumin in liposomes (free and encapsulated) was determined by lysing the liposomes using 6% v/v Triton X‐100 (Jose et al. 2018). Then, the concentration of free curcumin (in‐filtrate) and total curcumin were obtained by colorimetric method at a wavelength of 420 nm based on Equation (2) (Chaves et al. 2022).

(2) $$\text{Encapsulation efficiency (EE)}(\%)=\frac{\mathrm{concentration}\;\mathrm{of}\;\mathrm{encapsulated}\;\mathrm{curcumin}}{\mathrm{concentration}\;\mathrm{of}\;\mathrm{total}\;\mathrm{curcumin}}\times 100$$

### 2.5 Particle Size and Zeta Potential

The zeta potential of liposomes was measured by zeta sizer (Zeta Compact‐CAD France), and liposome size was determined by dynamic light scattering (DLS) (Particle Size Analyzer‐Cordouan‐Vasco3 France) (Vergara and Shene 2019).

### 2.6 Release

Curcumin release was measured using dialysis bags. The release medium was PBS containing ethanol (10%) and, Tween‐80 (0.5%). Nanoliposomal curcumin (8 mL) was poured into the dialysis bags and placed in flasks containing 240 mL of release medium. Two milliliters was withdrawn from the release medium at different times and replaced with fresh PBS solution. The absorbance of released curcumin was read by a spectrophotometer at 420 nm (R. Li et al. 2017).

### 2.7 Antimicrobial Properties

The serial dilution method was used to determine the MIC of free and nanoencapsulated curcumin (Wayne 2011). Dimethyl sulfoxide (DMSO) and 10% ethanol were used for diluting curcumin. In total, 190 μL of twofold dilutions of curcumin and nanocurcumin (2, 1, 0.50, 0.25, 0.125, 0.0625 mg/mL) were added to BHI in a 96‐well plate. In total, 10 μL of *S. mutans* overnight culture of bacterial suspension with a concentration of McFarland 0.5 was added to the wells. Culture medium without bacteria was used as negative control and chlorhexidine was used as antimicrobial control. After 24 h, ELISA reader was used to measure the turbidity at a wavelength of 600 nm. A difference of less than 0.5 in OD was considered as the MIC of curcumin (free and nanoencapsulated) against *S. mutans*. To obtain a more accurate MIC, the previous steps were repeated for concentrations between the first and second concentrations that did not cause turbidity.

### 2.8 Minimum Bactericidal Concentration

To measure minimum bactericidal concentration (MBC), 10 μL from transparent wells were transferred to BHI Agar and incubated for 24 h (37°C). The first concentration of free curcumin or nanoliposomal curcumin that did not grow a colony was considered as MBC (Karthikeyan et al. 2011).

### 2.9 Anti‐Biofilm Property of Curcumin

Different concentrations of curcumin and nanoliposomal curcumin (0, 0.125, 0.250, and 0.500 mg/mL) were prepared. The Amsterdam Active Attachment (AAA) model was used to investigate the effect of curcumin and nanoliposomal curcumin on inhibiting the growth of biofilms. Different concentrations of free and nanoliposomal curcumin, *S. mutans* (c180‐2), BHI, and 2% sucrose, were added to the wells of the 24‐well plate, and the lid of the model with 24 glass discs was placed on the plate. After 16 h of incubation at 37°C anaerobically, the biofilms were separated from the discs by ultrasound and transferred to BHI agar and incubated anaerobically for 72 h (Exterkate et al. 2010).

To evaluate the effect of curcumin on polymicrobial biofilms, various concentrations of free and nanoliposomal curcumin were added to the 24‐well plate along with Mc Bain's culture medium and saliva. The model's lid was placed on the plate and incubated anaerobically for 48 h at 37°C. The rest of the steps were done as mentioned for single‐species biofilms. TSA was used to plate diluted polymicrobial biofilms (Exterkate et al. 2010).

### 2.10 Lactic Acid Measurement

Before harvesting the biofilms from the glass discs, the model was placed on a new plate containing buffered peptone water and sucrose (0.2%). The plate was incubated for 3 h. The acid produced by biofilms was measured by the colorimetric method (Exterkate et al. 2010; Van Loveren et al. 2000). Equation (3), obtained from drawing the standard absorbance–lactic acid concentration curve, was used to calculate the concentration of lactic acid produced by biofilms.

(3) $$\text{OD}\;=0.1917C_{\mathrm{la}}\;+\;0.0142$$

*C*_la_ indicates the concentration of lactic acid.

### 2.11 Statistical Analysis

This research was conducted in the form of a completely randomized design. Comparison of means was done by one‐way ANOVA and Tukey's test at 99% confidence level with Minitab software version 18.0. Graphs were drawn with GraphPad Prism version 8.4.3.

## 3 Results

### 3.1 Properties of Nanoliposomes Loaded With Curcumin

The properties of nanoliposomes loaded with curcumin are listed in Table 1. The produced liposomes were within the nanoscale range and showed good dispersion, with PDI values indicating a narrow particle size distribution.

**Table 1:** Properties of nanoliposomes loaded with curcumin.

Properties	Nanoliposomal curcumin
Size (nm)	98.83 ± 2.89
PDI	0.168 ± 0.038
Zeta potential (mV)	−38.8 ± 2.47
Encapsulation efficiency (%)	64.37 ± 2.36

### 3.2 The Release of Curcumin From the Nanoliposome

**Figure 1:**
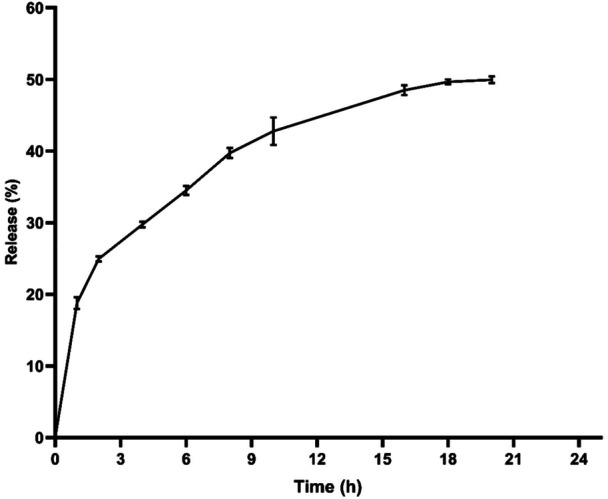
Curcumin release profile of liposomes in phosphate‐buffered saline containing ethanol (pH = 7). Results were reported as mean ± SD (*n* = 3).

The release of curcumin in PBS containing ethanol from the liposome was investigated for 24 h at 37°C (Figure 1). Less than 50% of curcumin was released from the liposome within 24 h.

### 3.3 MIC and MBC

MIC was determined to be 250 μg/mL for curcumin and 222 μg/mL for liposomal curcumin. The MBC of free curcumin was 2 mg/mL, but the MBC of curcumin nanoliposomes was 1 mg/mL. By nanoencapsulating curcumin, its MIC was reduced against *S. mutans*.

### 3.4 Effect of Curcumin on Single‐Species Biofilms

**Figure 2:**
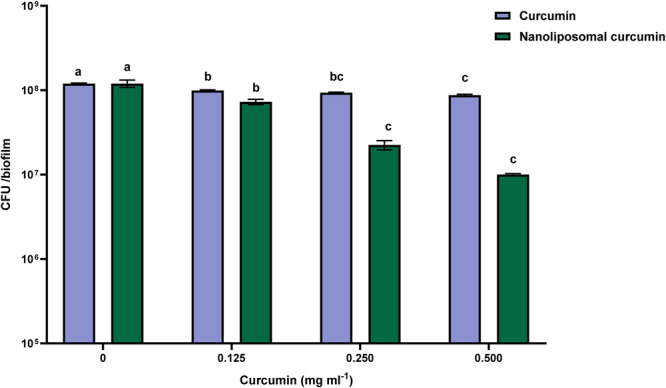
The CFU count of *S. mutans* in biofilms in the presence of different concentrations of curcumin (free and nanoliposomal). Results were reported as mean ± SD (*n* = 3). Means followed by the same letters in the same material were not significantly different (*p* > 0.01).

In Figure 2, the effect of curcumin (free and nanoliposomal) on the number of bacteria in the *S. mutans* biofilms can be observed. The most significant reduction of free curcumin was observed at the concentration of 0.500 mg/mL compared to the control treatment (*p* < 0.01). However, the decrease in the number of bacteria in biofilms was not significant compared to the concentration of 0.250 mg/mL (*p* > 0.01). Although statistically different concentrations create a considerable difference in the number of bacteria in biofilms (*p* < 0.01), the decrease does not reach a logarithmic cycle.

Anti‐biofilm effect of nanoliposomes containing curcumin was investigated. According to the Figure 2, reducing the CFU count in biofilms is more significant than the free curcumin at a concentration of 0.500 mg/mL. However, no significant difference was observed compared to the concentration of 0.250 mg/mL (*p* > 0.01). Although both free and nanoliposomal curcumin reduced the number of bacteria in biofilms, this decrease was more with nanoliposomal curcumin. It should be noted that DMSO and ethanol have been used to solubilize curcumin. Curcumin would be sediment in the wells if these substances were not used. Overall, these findings confirm that nanoencapsulation enhances the anti‐biofilm activity of curcumin against *S. mutans*, particularly at higher concentrations.

### 3.5 Effect of Curcumin on Polymicrobial Biofilms

**Figure 3:**
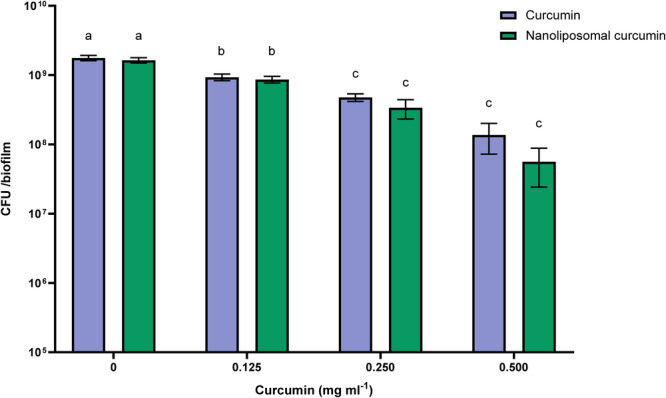
The CFU count of the poly biofilms in the presence of different concentrations of curcumin (free and nanoliposomal). Results were reported as mean ± SD (*n* = 3). Means followed by the same letters in the same material were not significantly different (*p* > 0.01).

The effect of free and nanoliposomal curcumin on polymicrobial biofilms can be seen in Figure 3. Liposomal curcumin at a concentration of 0.500 mg caused a decrease of about one logarithmic cycle of CFU count in biofilms, which was significant compared to the control sample (*p* < 0.01), but no significant difference was observed between the concentration of 0.500 and 0.250 mg/mL (*p* > 0.01). Free curcumin also decreased CFU count in polymicrobial biofilms, which difference was significant in all concentrations compared to the control sample (*p* < 0.01), but there was no significant difference between concentrations of 0.250 and 0.500 mg/mL (*p* > 0.01). Both free and liposomal curcumin reduced bacterial numbers in polymicrobial biofilms, with liposomal curcumin demonstrating greater efficacy at higher concentrations.

### 3.6 Acid Production

**Figure 4:**
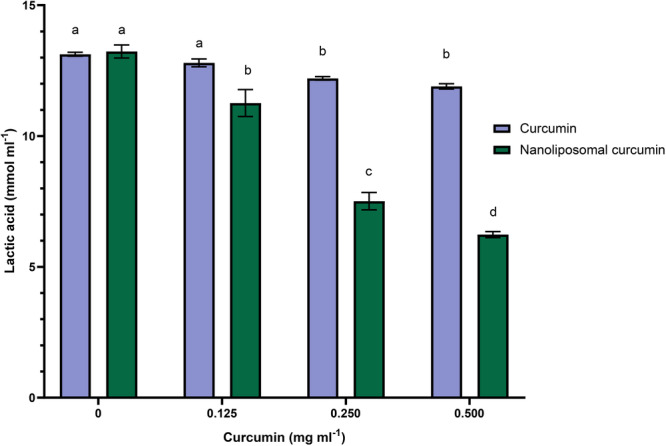
Lactic acid production by *S. mutans* biofilms. Results were reported as mean ± SD (*n* = 3). Means followed by the same letters in the same material were not significantly different (*p* > 0.01).

Another critical virulence feature of *S. mutans* biofilms is acid production and acid resistance, which are highly important when investigating natural products to prevent dental caries. According to Figure 4, free curcumin at 0.125 mg/mL did not significantly reduce acid production by *S. mutans* biofilms (*p* > 0.01). At 0.250 mg/mL and higher concentrations, however, a significant reduction was observed compared to the control (*p* < 0.01). Additionally, as the concentration of nanoliposome containing curcumin increased, the amount of acid produced decreased significantly (*p* < 0.01) so that the lowest concentration of lactic acid was observed at a concentration of 0.500 mg/mL.

**Figure 5:**
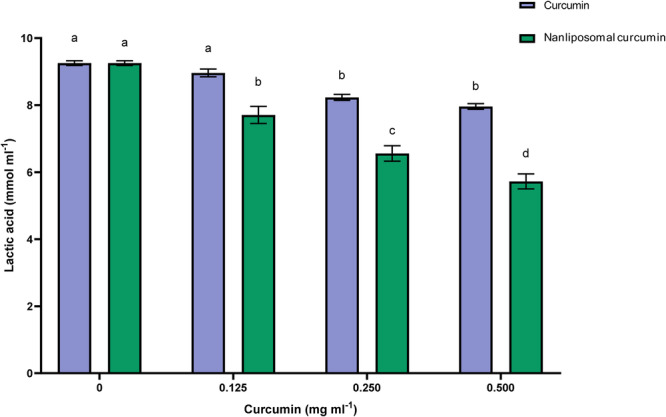
Lactic acid production by polymicrobial biofilms. Results were reported as mean ± SD (*n* = 3). Means followed by the same letters in the same material were not significantly different (*p* > 0.01).

The pattern of decreasing acid production produced by polymicrobial biofilms was almost the same as that of *S. mutans* biofilms, with the difference that acid reduction was observed to a lesser extent (Figure 5). Acid production in the presence of 0.125 mg/mL of free curcumin was not significant compared to the control sample (*p* > 0.01). At the concentration of 0.250, there was a significant difference compared to the control sample (*p* < 0.01). Despite the decrease in acid production in the concentration of 0.500 mL, this decrease was not significant compared to the concentration of 0.250 (*p* < 0.01). Nanoliposomal curcumin in all concentrations significantly reduced the amount of acid production by polymicrobial biofilms (*p* < 0.01). Nanoliposomal curcumin demonstrated stronger inhibition of acid production in both single‐species and polymicrobial biofilms compared to free curcumin, confirming its superior efficacy in reducing this critical virulence factor.

## 4 Discussion

### 4.1 Properties of Nanoliposomes Loaded With Curcumin

The nanoliposomes produced in our study were within the nanoscale range and showed good dispersion, with PDI values indicating a narrow particle size distribution. The word nano in nanotechnology refers to the scale of about 1–100 nm (Klabunde and Richards 2009), and PDI between 0.10 and 0.25 indicates a narrow particle size distribution (Vergara and Shene 2019).

### 4.2 The Release of Curcumin From the Nanoliposome

In our study, less than 50% of curcumin was released from liposomes, with a higher initial release likely due to curcumin molecules loosely associated with the liposomal surface. Other researchers have reported similar findings, with curcumin release from liposomes remaining below 60% (Chen et al. 2015; Pamunuwa et al. 2016; Tai et al. 2020).

### 4.3 MIC and MBC

In our study, the MIC and MBC of liposomal curcumin were lower than those of free curcumin. The MIC of free and encapsulated curcumin against *S. mutans* bacteria has been obtained by many researchers. However, the obtained MICs were different according to the curcumin extraction and microencapsulation methods. Mandroli and Bhat (2013) reported that the MIC of curcumin dissolved in DMSO against *S. mutans* bacteria was 333.33 µg/mL. Dwivedi and Vats (2020) found that the MIC of curcumin against *S. mutans* was 0.63 ± 0.02 mg/mL, while the MIC of the biofilm of this bacterium by curcumin was 0.057 ± 0.03 mg/mL. Hazzah et al. (2015) expressed that the MIC of curcumin against *S. mutans* bacteria was equal to 0.09375 mg/mL. Also, Song et al. (2012) investigated the ability of curcumin to inhibit the adhesion of *S. mutans* to the extracellular matrix and dental surfaces. They concluded that 128 μg/mL of curcumin could completely inhibit the growth of bacteria (Song et al. 2012).

Nanoformulations of curcumin have consistently shown enhanced antimicrobial activity compared to the free compound. Maghsoudi et al. (2017) reported MIC of chitosan, starch, and alginate nanoparticles against *S. mutans* bacteria as 0.114, 0.204, and 0.204 mg/mL, respectively. A significant decrease was observed in the MIC of nanocurcumin against other microorganisms compared to free curcumin (Bhawana et al. 2011; Jaiswal and Mishra 2018). According to the report of Pranjali et al. (2022), the concentration of liposomal curcumin, which had good antibacterial activity, was lower than the MIC of free curcumin. Zong et al. (2022) reported that curcumin nanoparticles had more solubility in water, enhancing the MIC and increasing the antimicrobial properties of curcumin, in particular against gram‐negative bacteria. It was reported by Targhi et al. (2021) that compared to free drugs, optimized nanoliposomal formulations enhanced MIC and MBC against bacteria. Xie et al. (2015) prepared curcumin nanoparticles by supercritical CO_2_, reporting higher solubility. Furthermore, the results revealed nanoparticles' better antimicrobial effect and their MIC were 50% lower than free curcumin solution (Xie et al. 2015).

It was observed that free and microencapsulated curcumin have antibiotic activity against *Bacillus subtilis* and *B. cereus* (Bhawana et al. 2011; Jaiswal and Mishra 2018; Rai et al. 2008; Wang et al. 2009). While these studies have reported different inhibitory concentrations for free curcumin against *B. subtilis*, they all showed a decrease in the MIC when nanocurcumin formulation was used instead of free curcumin (Bhawana et al. 2011; Jaiswal and Mishra 2018). However, this was not in line with Ding et al. (2018) findings since curcumin was trapped by cholesterol and lecithin, consequently increasing the MIC rate. Hence, all subsequent experiments were carried out at sub‐MIC concentrations (Ding et al. 2018). Our findings are consistent with the majority of previous reports, confirming that nanoencapsulation of curcumin reduces MIC and MBC values and enhances antimicrobial efficacy.

### 4.4 Effect of Curcumin on Single‐Species Biofilms

In the current study, both free and liposomal curcumin reduced CFU counts in *S. mutans* biofilms, with liposomal curcumin showing greater reduction. Findings are consistent with previous reports and confirm the enhanced efficacy of nanoencapsulation.

Curcumin could inhibit the Sortase A in *S. aureus* (Park et al. 2005). This enzyme is also responsible for the covalent bond and adhesion of cell surfaces to the cell wall, so it plays an essential role in biofilm formation. Curcumin is effective against sortase A activity and biofilm formation in *S. mutans* (Hu et al. 2013). Pandit et al. (2011) reported that curcuminoids have an inhibitory effect on *S. mutans* sucrose‐dependent adhesion to hydroxyapatite discs coated with saliva and acid production as well as on acid resistance of *S. mutans* biofilms. They concluded that the isolated fraction of curcuminoids and curcumin may be effective in reducing biofilms and consequently dental caries (Pandit et al. 2011). In a study, Song et al. (2012) concluded that curcumin against *S. mutans* has a potential anti‐adhesive effect on tooth surfaces and extracellular matrices. The anti‐adhesive effect of curcumin against *S. mutans* occurs on critical receptors for tooth decay (dental collagen) and infective endocarditis (fibronectin), respectively. According to the results, consumed curcumin could be used as an alternative method to prevent and treat dental diseases and infective endocarditis (Song et al. 2012). B. Li et al. (2018) concluded that after oral treatment with curcumin, *S. mutans* biofilms were reduced due to the reduction of extracellular polysaccharide production.

Nanoformulations have been highlighted as effective delivery systems for curcumin. Hazzah et al. (2015) reported that solid lipid nanoparticles provide an effective delivery system for curcumin. The possibility of more contact and interaction, increasing antimicrobial activity, and creating a delivery system with nano‐sized particles to create close contact between the drug and the microbial cell, in addition to curcumin stabilization, are some important effects of the presence of the mentioned material (Hazzah et al. 2015). Overall curcumin exhibits anti‐biofilm activity and nanoencapsulation further enhances these effects, highlighting liposomal curcumin as a promising formulation for controlling *S. mutans* biofilms and potentially reducing the risk of dental caries.

### 4.5 Effect of Curcumin on Polymicrobial Biofilms

In current study, both free and liposomal curcumin reduced CFU counts in polymicrobial biofilms, with liposomal curcumin at 0.500 mg/mL producing approximately one logarithmic cycle reduction compared to the control. These findings confirm the superior efficacy of liposomal curcumin and are consistent with previous reports on nanoformulations. Shahzad et al. (2015) reported that curcumin has an inhibitory effect on the growth of periodontopathic bacteria, such as *Porphyromonas gingivalis*, *Fusobacterium nucleatum*, and *Aggregatibacte actinomycetemcomitans*. However, the additional effects of curcumin on periodontopathic bacteria remain unclear (Shahzad et al. 2015). Karimi et al. (2019) concluded that liposome‐containing turmeric extract showed higher antibacterial activity against all investigated microorganisms compared to free turmeric extract.

Nanocurcumin has consistently demonstrated enhanced antimicrobial activity. Gopal et al. (2016a) compared the antibacterial properties of curcumin at macro‐, micro‐, and nanoscales and reported that the nanoscale showed higher antibacterial properties against pathogenic bacteria. Bhawana et al. (2011) showed that nanocurcumin dispersed in water more effectively than free curcumin and exhibited stronger activity against *Aspergillus niger*, *Escherichia coli*, *Pseudomonas aeruginosa*, *B. subtilis*, and *S. aureus*, with Gram‐positive bacteria being more sensitive. They found that curcumin nanoparticles could enter the bacterial cell by breaking the cell wall, which leads to cell death (Bhawana et al. 2011).

The inhibition of in vitro growth of methicillin‐resistant *S. aureus* (MRSA) and *P. aeruginosa* in a dose‐dependent manner was reported by Krausz et al. (2015) after synthesizing curcumin‐loaded chitosan nanoparticles. Plankton growth of both microorganisms and MRSA cell damage may be inhibited by synthesized nanoparticles (Krausz et al. 2015). Cho et al. (2020) prepared nanoemulsions containing *Curcuma xanthorrhiza* oil. *C. xanthorrhiza* is extracted from the turmeric plant, which has strong antimicrobial effects. *C. xanthorrhiza* is very hydrophobic and often DMSO is used as a solvent, which is toxic and dangerous for young children. Therefore, they prepared a nanoemulsion using *C. xanthorrhiza*, water, and polysorbate 80. According to the results, turmeric extract has a strong antimicrobial effect (Cho et al. 2020).

Gopal et al. (2016a) compared the antimicrobial activity of nanocurcumin with conventional curcumin. The results of the CLS microscope showed an increase in the entry of nanocurcumin particles into microbial cells. They also reported that the nature of nanocurcumin's nanostructure leads to increased cell interaction and, as a result, its greater efficiency in destroying microbial cells in the mouth. In addition, the solubility of nanocurcumin was an essential factor in improving its antimicrobial activity (Gopal et al. 2016b).

Curcumin liposomes significantly inhibited the formation of biofilm at sub‐MIC concentration. Under treatment with curcumin liposomes, *Aeromonas hydrophila* and *Serratia grimesii* biofilms content, respectively, decreased by 65.80% and 80.14%. Compared to the control, a concentration of 0.3 mg/mL shows the effective control of biofilms of foodborne pathogens by curcumin liposomes (Ding et al. 2018).

These findings align with our results, where liposomal curcumin demonstrated stronger inhibition of polymicrobial biofilms compared to free curcumin.

### 4.6 Acid Production

In our study, free curcumin reduced acid production in *S. mutans* biofilms only at concentrations of 0.250 mg/mL and above. In contrast, nanoliposomal curcumin caused a clear, dose‐dependent decrease (*p* < 0.01). A similar pattern was observed in polymicrobial biofilms, confirming the superior efficacy of liposomal curcumin in reducing acid production compared to free curcumin.

Previous studies have reported that curcuminoids had a decreasing effect on acid production and acid resistance of *S. mutans* biofilms, which may be due to the effect of these extracts on bacterial glycolytic pathways. Some natural products could disrupt the membrane's proton motive force and inhibit membrane‐related enzymes, which have a relationship with glycolytic enzymes, sugar transport, and the expression of metabolism‐related specific proteins in *S. mutans*, which affects the acid production *S. mutans* (Pandit et al. 2011).

It has been reported that concentrations of 0.5–4 mg/mL of turmeric essential oil could inhibit the acid production and growth of *S. mutans*. Also, turmeric essential oil has a significant ability to inhibit the adhesion of *S. mutans* to saliva‐coated hydroxyapatite and could prevent the formation of *S. mutans* biofilms (Lee et al. 2011).

Nanoencapsulation further enhances the efficacy of antimicrobial substances. Formulations of antimicrobial substances in liposomes could improve their inhibitory properties against *S. mutans* biofilms and reduce acid production (Habibi et al. 2022). Liposomal curcumin appears to be a promising option for controlling acid production in biofilms.

## 5 Conclusions

Antibacterial and anti‐biofilm properties of curcumin have been reported by other researchers. However, due to the limitation of using curcumin caused by its hydrophobicity, it is necessary to investigate methods that can increase the solubility of curcumin. According to our findings, nanoliposomal curcumin had lower MIC than free curcumin against *S. mutans*. In addition, nanoliposomal curcumin reduced CFU count in single‐species and polymicrobial biofilms. Acid production by biofilms is very important due to its effect on tooth demineralization. Nanoliposomal curcumin decreased acid production in both single‐species and polymicrobial biofilms. In general, it was found that nanoencapsulation of curcumin in liposome improved its antibacterial, anti‐biofilm, and inhibition of acid production by oral biofilms. It should be noted that this study used only one clinical sample, which may limit the generalizability of the findings. Further studies with multiple isolates and larger sample sizes are needed to confirm the broader applicability of the results.

## Author Contributions

**Parisa Habibi:** writing – original draft, formal analysis, investigation. **Farideh Tabatabaei Yazdi:** project administration, supervision, conceptualization, methodology. **Seyed Ali Mortazavi:** supervision, conceptualization, methodology. **Mohammad Morad Farajollahi:** resources, methodology. All authors provided critical feedback and helped shape the research, analysis, and manuscript.

## Ethics Statement

The study was approved by the ethics committee of the Ferdowsi University of Mashhad (document number: IR.UM.REC.1400.368).

## Conflicts of Interest

The authors declare no conflicts of interest.

## Data Availability

The data that support the findings of this study are available from the corresponding author upon reasonable request.
